# Examining resting-state network connectivity in children exposed to perinatal maternal adversity using anatomically weighted functional connectivity (awFC) analyses; A preliminary report

**DOI:** 10.3389/fnins.2023.1066373

**Published:** 2023-03-16

**Authors:** Sondos Ayyash, Aleeza Sunderji, Heather D. Gallant, Alexander Hall, Andrew D. Davis, Irina Pokhvisneva, Michael J. Meaney, Patricia Pelufo Silveira, Roberto B. Sassi, Geoffrey B. Hall

**Affiliations:** ^1^School of Biomedical Engineering, McMaster University, Hamilton, ON, Canada; ^2^Department of Psychology, Neuroscience & Behaviour, McMaster University, Hamilton, ON, Canada; ^3^Department of Psychiatry and Behavioural Neurosciences, McMaster University, Hamilton, ON, Canada; ^4^Ludmer Centre for Neuroinformatics and Mental Health, Douglas Mental Health University Institute, McGill University, Montreal, QC, Canada; ^5^Faculty of Medicine and Health Sciences, Department of Psychiatry, McGill University, Montreal, QC, Canada; ^6^Translational Neuroscience Program, Agency for Science, Technology and Research (A*STAR), Singapore Yong Loo Lin School of Medicine, Singapore Institute for Clinical Sciences and Brain – Body Initiative, National University of Singapore, Singapore, Singapore; ^7^Department of Psychiatry, The University of British Columbia, Vancouver, BC, Canada

**Keywords:** middle childhood, perinatal adversity, resting-state network (RSN), functional connectivity (FC), structural connectivity (SC), neurodevelopment

## Abstract

**Introduction:**

Environmental perturbations during critical periods can have pervasive, organizational effects on neurodevelopment. To date, the literature examining the long-term impact of early life adversity has largely investigated structural and functional imaging data outcomes independently. However, emerging research points to a relationship between functional connectivity and the brain’s underlying structural architecture. For instance, functional connectivity can be mediated by the presence of direct or indirect anatomical pathways. Such evidence warrants the use of structural and functional imaging in tandem to study network maturation. Accordingly, this study examines the impact of poor maternal mental health and socioeconomic context during the perinatal period on network connectivity in middle childhood using an anatomically weighted functional connectivity (awFC) approach. awFC is a statistical model that identifies neural networks by incorporating information from both structural and functional imaging data.

**Methods:**

Resting-state fMRI and DTI scans were acquired from children aged 7–9 years old.

**Results:**

Our results indicate that maternal adversity during the perinatal period can affect offspring’s resting-state network connectivity during middle childhood. Specifically, in comparison to controls, children of mothers who had poor perinatal maternal mental health and/or low socioeconomic status exhibited greater awFC in the ventral attention network.

**Discussion:**

These group differences were discussed in terms of the role this network plays in attention processing and maturational changes that may accompany the consolidation of a more adult-like functional cortical organization. Furthermore, our results suggest that there is value in using an awFC approach as it may be more sensitive in highlighting connectivity differences in developmental networks associated with higher-order cognitive and emotional processing, as compared to stand-alone FC or SC analyses.

## 1. Introduction

Resting-state fMRI is a neuroimaging technique that has been used to map functional brain networks comprised of cortical and subcortical regions (Lee et al., 2013) when an individual is at rest. A measure of functional connectivity (FC) can be derived from rsfMRI. FC refers to the temporal correlation between brain activity in spatially distinct regions. It is commonly assumed that regions belong to the same network if their functional activity is correlated. During middle childhood, resting state networks (RSNs) have not reached full maturity and are continually undergoing constant changes (i.e., reorganization, strengthening/weaking of connections, segregation/integration) (Fair et al., 2009; Rosenberg et al., 2020). Network maturation reflects functional specialization as neural assemblies and regions that make up functional cortical subunits become increasingly domain-specific and there is a shift from engaging diffuse regions to more focal brain activation patterns (Casey et al., 2005; Durston et al., 2006). Thus as development proceeds there is a shift from more diffuse less functionally specialized regions assembled in short-range circuits to more functionally specialized focal regions that are more broadly distributed. This configuration is particularly important for complex, “higher-order” cognitive capacities because it provides for the synchronization of processing across functionally specialized regions that are distal to one another (Reijneveld et al., 2007; van Meel et al., 2012). Increases in cortico-cortical connectivity as opposed to strong and abundant subcortico-cortical connectivity are also observed (Supekar et al., 2009). However, temporally correlated activity does not provide an indication of direct anatomical connections between brain regions.

To examine structural connectivity (SC), DTI is an MRI technique that identifies white matter pathways, non-invasively (Fernandez-Miranda et al., 2012). Structural connectivity refers to the presence of physical white matter pathways that connect brain regions and can be measured using DTI metrics such as fractional anisotropy (FA). Across childhood and adolescence cortical white matter increases linearly, and reflects changes in myelination during this period (Sowell et al., 2003). As the children in our cohort are now in middle childhood, we are presented with a unique opportunity to study how brain changes in structure and function relate to the acquisition of emergent developmental capacities.

Evidently, FC and SC capture distinct but interrelated properties of the brain-network connectome (Suárez et al., 2020). Importantly, the trajectory of functional connectivity within brain networks may be mediated by ongoing structural developmental processes (i.e., myelination, pruning, etc.) (Jolles et al., 2011; Huang et al., 2015). However, there is an imperfect correspondence between structural and functional connectivity (Damoiseaux and Greicius, 2009; Suárez et al., 2020). While strong SC is predictive of strong resting-state FC between regions, the reverse inference is not as reliable (Damoiseaux and Greicius, 2009; Honey et al., 2009). As such, neither functional nor structural techniques alone can reveal a complete picture of brain maturation (Park and Friston, 2013), thereby warranting the study of how functional and structural connectivity interact during development. Previous research has suggested that a multimodal approach, combining parameters of DTI-based structural connectivity and fMRI-based resting-state functional connectivity to study development would make more effective use of information in MR scans as opposed to performing each technique independently (Straathof et al., 2020).

Accordingly, in this study we employ a novel, multimodal imaging approach that examines functional and structural connectivity concurrently. Our pipeline uses a neuroimaging toolbox in combination with a mathematically dense approach for fusing fMRI and DTI metrics into a single combined measure. The neuroimaging toolbox applied in this study is known as Functional And Tractographic Analysis Toolbox (FATCAT) and consists of a set of AFNI commands that are publicly available for processing MRI data. The second component of the pipeline, the anatomically weighted functional connectivity (awFC) model, fuses FC and SC measures into a single unit identified as the awFC metric. Connectivity is measured differently by each modality. For instance, while structural connectivity counts the number of tracts, functional connectivity measures temporally correlated regions. By combining structural and functional dissimilarity multiplicatively, the awFC merges structural and functional connectivity in a modality-independent manner. The awFC metric can provide a quantitative measure of the combined impact of structural and functional connectivity on brain networks, rather than studying the connectivity from one perspective (one modality) alone. According to Rebeiro and colleagues (2017), their proposed approach of multiplying both structural and functional connectivity data can generate a hybrid connectivity matrix that enables comprehensive analysis of brain networks. As a result, combining data from many modalities can theoretically lead to the provision of a more reliable inference on the “connectome” (Liu et al., 2018). The awFC metric we describe, measures the combined effect of connectivity on brain networks. Given that the macroscopic connectome is a blend of structural and functional connectivity, combining structural and functional connection in a data fusion approach may provide a better representation of the complex human connectome. While the FATCAT-awFC pipeline was first introduced in Ayyash et al. (2021), here we explore the utility in applying a data fusion approach to study brain changes associated with development. This article extends our previous work by applying the method to study brain connectivity within RSNs in a sample of children aged 7–9 years old.

Adverse maternal experiences early in a child’s life can have pervasive effects across the domains of cognition, emotion, and behavior (Glover, 2011; Monk et al., 2012; Adamson et al., 2018; Van den Bergh et al., 2020). It is possible that early life experiences bias the allocation of resources toward neural systems that are critical for effective adaptation within the given environmental context (Chelini et al., 2022). For instance, neurodevelopmental patterns which favor emotional responses (e.g., hypervigilant limbic system, improved threat perception, stronger sympathetic activation, etc.) may give rise to maladaptive behavioral responses, and potentially establish a footing for poor psychiatric outcomes (Chelini et al., 2022). Therefore, while the precise underlying mechanisms remain unclear, alterations in structural and functional neurodevelopment are proposed as putative pathways by which adversity may become biologically embedded and transmit its effects across generations (Van den Bergh et al., 2020). When studying the link between early adversity and subsequent neurodevelopmental outcomes, the timing of adversity exposure must be considered. Prenatal and postnatal periods of development are the most rapid phases of brain development during which formative processes such as neurogenesis, migration, differentiation, myelination, and synaptogenesis are occurring to establish the baseline framework for future development (Stiles and Jernigan, 2010). In early postnatal life, exuberant axonal removal also occurs to establish the number of connections that comprise the structural connectome (Collin and van den Heuvel, 2013). At this time, there exists many sensitive periods of development which are characterized by heightened plasticity and vulnerability to the environment (Nelson and Gabard-Durnam, 2020). It follows that environmental insults during these early periods of development can interrupt normative developmental processes and leave lasting effects on development. As posited by proponents of the prenatal programming hypothesis, the in-utero environment provides cues to the fetus about its expected postnatal environment (Pluess and Belsky, 2011). Upon exposure to adverse prenatal conditions, fetal brain development can calibrate in a manner that supports optimal functioning within an expected adverse postnatal environment (Glover, 2011; Pluess and Belsky, 2011).

Emerging research continues to support the idea that there is immense potential for perturbations in the prenatal environment to redirect development. For instance, poor maternal mental health during pregnancy is linked to atypical emotional and cognitive development in the developing child. Children whose mothers experienced anxiety or depression during pregnancy are at increased risk for mental illnesses such as anxiety (O’Connor et al., 2002; Van den Bergh and Marcoen, 2004; Bergman et al., 2007), attention deficit hyperactivity disorder (ADHD) (Huizink et al., 2002; O’Connor et al., 2002; Van den Bergh and Marcoen, 2004), and conduct disorder (Barker and Maughan, 2009). These outcomes are likely a consequence of modifications in neurodevelopment. De Asis-Cruz et al. (2020) report that prenatal maternal anxiety is associated with increased functional connectivity between earlier developing brain regions associated with arousal and salience (e.g., brainstem and sensorimotor areas) and reduced functional connectivity between regions that subserve higher-order cognitive functions (i.e., executive control and default mode network regions). These alterations in child neurodevelopment are identifiable as early as the second trimester of pregnancy. A study exploring resting-state functional connectivity in infants aged 6 months also noted that prenatal maternal depression is associated with altered connectivity between the amygdala and other brain regions involved in emotion generation and regulation such as the temporal cortex, insula, ventromedial prefrontal cortex, medial orbitofrontal cortex, and anterior cingulate cortex (Qiu et al., 2015). Posner et al. (2016a) observed atypical structural and functional connectivity in the amygdala-dorsal prefrontal cortex circuity amongst infants exposed to prenatal maternal depression. Overall, such evidence points toward the propensity of maternal mental health to affect subsequent child neurodevelopment, especially in regions critical for salience detection and emotion regulation.

Since adverse experiences are likely to exhibit continuity across time, it is critical to account for both prenatal and postnatal mental health experiences (O’Connor et al., 2002). Poor postnatal maternal mental health can interfere with the capacity for sensitive and responsive caregiving, affecting the quality, and nature of the child’s early environment. Reductions in the amount of social and emotional stimulation and can have pervasive effects on subsequent child development (Goodman et al., 2011; Tottenham, 2015). For instance, functional neuroimaging studies have repeatedly shown associations between postnatal depressive symptoms and altered functional connectivity between limbic regions and mesocortical and mesolimbic networks (Wang et al., 2020; Cattarinussi et al., 2021), reshaping the capacity for emotion regulation and reward learning. Structurally, diffusion tensor imaging (DTI) investigations have revealed that children exposed to prenatal or postnatal maternal depression exhibit microstructural alterations in limbic regions, prefrontal areas, the cingulum, corpus callosum, and fornix (Lebel et al., 2016; Cattarinussi et al., 2021). The directionality of changes in diffusivity remains inconsistent for the amygdala and frontal regions however, reduced anisotropy and increased diffusivity in the cingulum is often reported (Cattarinussi et al., 2021). Maternal anxiety during pregnancy is negatively associated with fractional anisotropy in prefrontal regions, the middle frontal gyrus, and fornix (Graham et al., 2020). Ultimately, these changes in white matter connectivity may underlie difficulties observed in executive functioning, attention, and emotion regulation.

Adverse experiences tend to co-occur (Sheridan and McLaughlin, 2014). As demonstrated by the adverse childhood experiences (ACE) study, there exists a strong relationship between the number of adverse childhood exposures and the number of long-term health risk factors, suggesting that the effect of ACEs on long-term health may be robust and cumulative (Felitti et al., 2019). Accordingly, it is recommended that research considers the impact of unique combinations of adverse exposures on long-term health outcomes (Felitti et al., 2019). Several studies have reported higher rates of poor maternal mental health in low socioeconomic contexts (Beeghly et al., 2003; Goyal et al., 2010; Pitchik et al., 2020; Radey and McWey, 2021). Therefore, in the present work, we explore how early life exposure to poor maternal mental health and socioeconomic status affects child neurodevelopment years later. Socioeconomic status (SES) can be regarded as one’s access to material and social resources (e.g., nutrition, housing, safe neighborhoods, income, and education). SES-related health disparities pose a risk for the physical and mental health and wellbeing of a mother during pregnancy as well as her child. In fact, SES reportedly moderates the impact of poor maternal health on offspring outcomes (Stein et al., 2008, 2014; Pearson et al., 2013). Postnatally, a low SES in childhood can set constraints on the quality of learning experiences that the child gains as he/she engages with their environment. It has been found that the risk of developing mental health conditions (e.g., ADHD, externalizing problems, depression, substance use, schizophrenia, etc.) over the lifespan is two to three times higher for those from a low SES background (Wadsworth and Achenbach, 2005). Deficits in language development and executive functioning are commonly reported outcomes linked to SES (Hackman and Farah, 2009; Pace et al., 2017). Functional neuroimaging studies also report evidence of altered neural activation in regions associated with language processing in children from lower SES backgrounds (Raizada et al., 2008; Romeo et al., 2018). In neonates, infants, and children, SES is associated with altered maturation in neural networks underlying sensory, emotional, and executive functioning (Gao et al., 2015; Turesky et al., 2019; Ramphal et al., 2020).

Overall, evidence of atypical brain connectivity in the offspring of mothers who experienced pre or postnatal adversity has been demonstrated through fMRI and DTI studies (Rifkin-Graboi et al., 2015; Posner et al., 2016a; Dean et al., 2018). However, to date, there remains limited research exploring the impact of maternal adversity on structural and functional maturational particularly during the middle childhood period. Movement and low compliance with task demands pose obstacles in using MRI to study development within this age group, thereby hampering progress in our understanding of brain maturation. Here we apply stringent criteria (Power et al., 2012) for movement during rsfMRI; opting for improved data quality at a cost to study sample size. We acknowledge, the limitations imposed by these steps, however, as an initial examination of our data, we were interested in understanding whether the predicted alterations in FC and SC associated with maternal adversity exposure were captured best using a multimodal data fusion approach. As such, in the following study, we concurrently study structural and functional neurodevelopment using resting-state functional magnetic resonance imaging (rs-fMRI) and diffusion tensor imaging (DTI) within a sample of 7–9-year-old children whose mothers experienced adversity during the prenatal and/or postnatal period and a sample of similarly matched healthy control children. We hypothesized that children of mothers who experienced adversity will exhibit greater awFC between regions in RSNs in comparison to controls. As early adversity is associated with aberrant white matter (Lebel et al., 2016; Demers et al., 2021b) and gray matter development (Lupien et al., 2011; Callaghan and Tottenham, 2016; Demers et al., 2021a), we suggest that examining both features of development in one comparison may be more informative to our understanding of the relation between perinatal maternal adversity and child neurodevelopment. We acknowledge that early life environmental experiences may influence the allocation of resources in favor of developing systems that are critical for adaption within a certain environmental context (Chelini et al., 2022).

## 2. Materials and methods

### 2.1. Sample

Our sample consisted of mother-infant dyads from the MAVAN (Maternal Adversity, Vulnerability, and Neurodevelopment) cohort—a longitudinal birth cohort from Hamilton, Ontario and Montreal, Quebec. Beginning from 6 months, children were assessed using behavioral, cognitive, and diagnostic tools. Information regarding prenatal and postnatal experiences were acquired from mothers. Inclusion criteria included mothers who were above the age of 18, delivered a singleton pregnancy, and fluent in English or French. Exclusion criteria entailed mothers with a history of incompetent cervix, presence of placenta previa, maternal severe chronic illness, impending delivery, or a fetus affected by a major anomaly. Ethics approval was obtained from Hamilton Integrated Research Board and the Douglas Mental Health University Institute. Informed consent was acquired from all participants. Compensation of $25 was provided for every visit.

Functional magnetic resonance imaging and DTI data was available for 33 MAVAN children in the middle childhood age group and thus, analyses were conducted using this subsample. Excluded from analysis were children with head motion that exceeded a relative mean displacement of 0.55 mm. This stringent threshold was considered an important criterion given our sample age and resulted in the loss of eight children from the sample (see Satterthwaite et al., 2012). Scans that had missing resting-state fMRI data points (*n* = 2), rsfMRI artifacts (signal inhomogeneity) (*n* = 1), or missing DTI data (*n* = 5) were omitted from the analysis. Our final sample included a total of 17 subjects (11 females, 6 males)—9 children whose mothers did not have a history of prenatal and/or postnatal adversity (7 females, 2 males) and 8 children with history of maternal adversity (4 females, 4 males). Children were between the ages of 7 and 9 years old (mean = 7.63 years; *SD* = 0.66).

### 2.2. Maternal adversity score

Maternal adversity is represented by a composite binary adversity score which accounts for perinatal maternal mental health (i.e., anxiety and/or depression) as well as socioeconomic status. This composite score was generated by researchers at the Women’s Health Concerns Clinic in Hamilton, Ontario. Maternal depression was measured using the Edinburgh Postnatal Depression Scale (EPDS) and the Montgomery-Asberg Depression Rating Scale (MADRS). The EPDS is a 10-item, self-report questionnaire that provides an indication of depressive symptomatology during the perinatal period (Cox and Holden, 2003). The MADRS is a 10-item clinician-rated scale assessing symptom severity if depression is suspected (Montgomery and Asberg, 1979). Maternal anxiety was probed using the State-Trait Anxiety Inventory (STAI) and the Hamilton Anxiety Rating Scale (HAM-A). The STAI is a self-report measure of trait (general feelings) and state (momentary feelings) anxiety (Spielberger, 1983). The HAM-A is a 14-item, clinician-report measure that assesses the severity of anxiety symptoms. If a mother achieved an EPDS score >11 or a STAI score >29 and HAM-A score >17, or MADRS score >6, they were classified as experiencing perinatal depression and/or anxiety. Low socioeconomic status was declared if the parental income met or fell below the low-income tax cut-off level (i.e., ∼$30,000). Prenatal assessments were collected between 12 and 36 weeks and postnatal assessments were obtained within the first postnatal year.

One point was assigned for the presence of perinatal depression and/or anxiety and/or low SES status. For group analyses, children with a maternal adversity score above 1 were assigned to the adversity group, while those with a score of 0 were placed in the healthy control group.

### 2.3. Magnetic resonance imaging

In a separate visit prior to MRI acquisition, children were invited for a mock scanning session to get familiarized with the scanning environment. Magnetic resonance images were acquired using a GE Discovery 750 3T MR scanner (General Electric Healthcare, Milwaukee, WI) with a 32-channel head coil at the Imaging Research Centre, St. Joseph’s Healthcare (Hamilton, Canada), and a 3T Siemens Trio scanner (Siemens, Erlangen, Germany) at the Cerebral Imaging Center, Douglas Research Centre (Montreal, Canada).

Functional images were obtained using a single-shot gradient-echo echo-planar imaging (EPI) and the acquisition parameters included: TE (echo time) = 35 ms, TR (repetition time) = 3,000 ms, flip angle = 90, matrix = 64 × 64 × 45, voxel size = 3.75 mm × 3.75 mm × 3 mm, interslice gap = 0, slice thickness = 3 mm, FOV = 24 cm^2^, 108 volumes (scan time 5.4 min), ascending interleaved sequence. Diffusion tensor images were acquired with a single-shot spin-echo EPI sequence, with 66 gradient directions at 1,000 s/mm^2^. Additional DTI parameters included: *TE* = 87 ms, *TR* = 8,800 ms, matrix = 122 × 122 × 70, voxel size = 2 mm × 2 mm × 2 mm, FOV = 24.4 cm^2^, 70 ascending interleaved slices, slice thickness = 2 mm (no slice spacing). Three non-diffusion weighted b = 0 s/mm^2^ images were also collected.

Magnetic resonance imaging scanning was well-tolerated, and positive feedback was provided by the parents, including indications of willingness to participate in future studies.

## 3. Analyses

### 3.1. Functional magnetic resonance imaging pre-processing and motion correction

Resting-state functional data were pre-processed using FSL version 6.0.1 (Jenkinson et al., 2012), using standard protocols as described in prior analyses (Krafft et al., 2014). Pre-processing steps included: (1) discarding the first three volumes of every participant’s functional data to account for magnetic field homogenization, (2) interleaved slice timing correction, (3) brain extraction toolbox (BET) (Smith, 2002) for skull stripping, (4) motion correction using *MCFLIRT* (Saccà et al., 2018), (5) spatial smoothing using a Gaussian kernel with FWHM = 5 mm, and (6) high pass filtering (0.1 Hz). Functional data was normalized and registered to standard MNI152 space (12 DOF) and resampled to a 4-mm cubic voxel for subsequent analysis.

An additional step was introduced in the standard pre-processing pipeline (Goto et al., 2016), which involves despiking the functional data using AFNI (version 18.2.15) (Patel et al., 2014). This method is said to be more effective than other motion correction methods such as scrubbing (Goto et al., 2016).

A standard adult functional brain template (MNI 152) was used for registration and normalization. Previous studies by Muzik et al. (2000) and Wilke et al. (2002) demonstrated that spatial registration of child data aged 5 and above and 6 and above, respectfully, to adult brains, are acceptable with negligible or minor distortions (if any). Additionally, children over the age of five do not undergo significant increases in brain volume (Giedd et al., 1996; Casey et al., 2000).

Head motion is a major source of artifacts in connectivity studies (Power et al., 2012), and thus, it is necessary to exclude subjects with gross motion (Satterthwaite et al., 2012). Satterthwaite et al. (2012) deemed gross motion in functional data as having a relative mean displacement greater than 0.55 mm. Therefore, subjects with an average relative volume-volume displacement greater than 0.55 mm were excluded from our study (*n* = 8). While our final sample size was impacted by this criterion, it is important to be stringent on motion parameters.

Five RSNs were examined: the default mode network (DMN), involved in internally generated thoughts, the limbic network (LIM), participating in emotion regulation, and the ventral attention network (VAN), dorsal attention network (DAN), and frontal parietal network (FPN), all of which subserve higher-order cognitive functions. Previous literature has reported that connectivity in these RSN’s has been impacted upon maternal adversity exposure (Van den Bergh et al., 2018).

### 3.2. Functional magnetic resonance imaging analysis using FATCAT

Group independent component analysis (GICA) was applied to the resting data using MELODIC (FMRIB Analysis Group, Oxford University; Beckmann and Smith, 2004; Beckmann et al., 2005). The pre-processed functional data in MNI space was passed through the MELODIC GUI with the component number set at 20 and the decomposition approach set to multisession temporal concatenation. Independent components were compared and matched to the standard Yeo seven network template (Yeo et al., 2011) using a spatial cross correlation from FATCAT’s “*3dMatch*” command (Taylor and Saad, 2013). Of the 20 components generated, five networks were consistent with standard RSNs (with a mean correlation of *r* = 0.62): DMN, FPN, LIM, VAN, and DAN. The remaining maps were either taken to be artifactual, noise or simply not matching with the components. Each of the independent component Z-score maps for the five identified networks were further segmented at the group level into discrete regions of interest (ROIs) using FATCATs “3dROIMaker” with an empirically determined threshold for each network, in order to use the ROIs with the diffusion data, the ROIs were transformed into diffusion-weighted space and inflated using FATCAT’s “*3dROIMaker”* command to overlap or abut white matter tracts. The Pearson correlation coefficient was calculated between the mean time courses of each ROI pair, using the non-inflated ROIs with FATCAT’s “*3dNetCorr”* command (Taylor and Saad, 2013). Functional connectivity (Pearson’s correlation) was estimated for each subject. Functional connectivity group differences were then investigated using a Wilcoxon-test between the adversity exposed and control groups using R code and corrected for multiple comparisons (which will be discussed in greater detail later in this section). Statistically significant (*p*_*adj*_ < 0.05) group differences were identified and reported in this study as “conventional functional connectivity.”

### 3.3. Diffusion tensor imaging pre-processing

Diffusion tensor imaging pre-processing steps were performed using a source-code repository (Davis et al., 2019). Pre-processing involved a combination of FSL and AFNI commands and consisted of the following steps: (1) Diffusion-weighted images and *b* = 0 images were converted from DICOM to NIFIT using dcm2nii, (2) Eddy current distortions and motion were corrected (registered to *b* = 0 reference volume) with FSL’s “*eddy_correct”* command (Jenkinson et al., 2012) and diffusion vectors were rotated, (3) DTI images were skull stripped using FSL’s “BET” (Smith, 2002) (4) FATCAT’s “*3dDWItoDT*” (Taylor and Saad, 2013) was applied for diffusion tensor fitting, and FA maps were generated (in diffusion-weighted space), (5) FA maps were spatially normalized to a standard FA template (FMRIB58) for group analysis.

### 3.4. Diffusion tensor imaging analysis using FATCAT

Uncertainty maps from FA and principal eigenvector were generated, with the FATCAT command “*3dDWUncert”* (Taylor and Saad, 2013) to include in probabilistic tractography. Probabilistic tracking was then estimated between inflated ROI-pairs using FATCAT’s “3dTrackID” (Taylor and Saad, 2013) with the default standard settings: *FA* = 0.2, turning angle = 60, Monte Carlo iterations = 1,000. The DTI measures, such as the distance and number of tracts between each inflated group-level ROI pair were estimated for each subject. “Conventional structural connectivity” was calculated by counting the number of tracts between two ROIs (a DTI metric output by FATCAT). Group-level comparison of structural connectivity was performed to study differences between adversity and control groups. Between-group comparisons were performed with a Wilcox-test and corrected for multiple comparisons using the false discovery rate. Comparisons that were statistically significant (*p*_*adj*_ < 0.05) are reported in this study and discussed.

### 3.5. Combined structural and functional connectivity analysis using the awFC method

**FIGURE 1:**
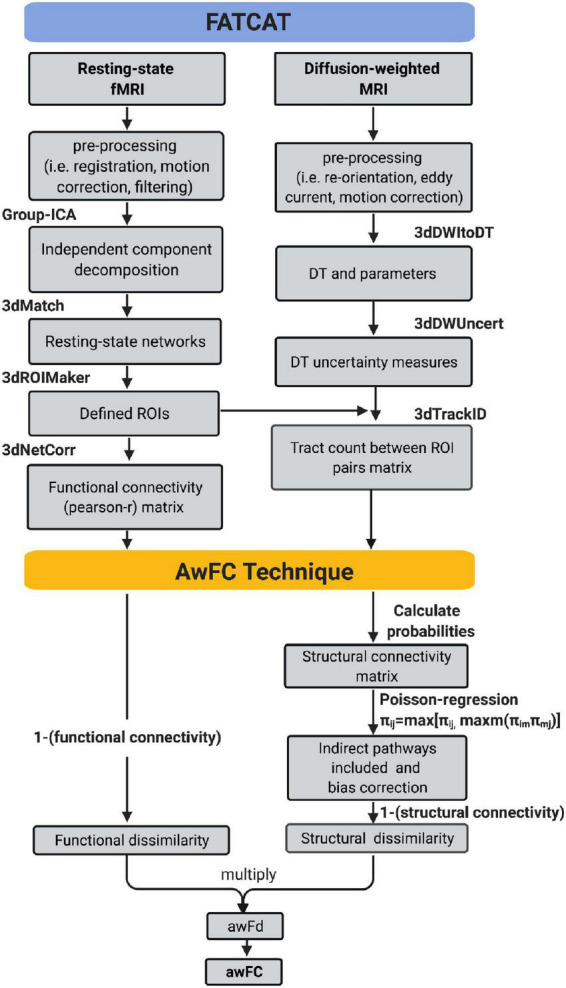
Functional And Tractographic Analysis Toolbox-awFC pipeline. This is a two-stage pipeline and when combined, results in a more straightforward approach for combining fMRI and DTI data. The first stage is the “Functional and Tractographic Connectivity Analysis Toolbox” (FATCAT) pipeline. The outputs include functional connectivity (derived from fMRI) and the number of tracts (derived from DTI). The second stage of the pipeline is known as the anatomically weighted functional connectivity (awFC), which processes the output of FATCAT to produce the anatomically weighted functional connectivity measure (awFC measure). fMRI, functional magnetic resonance imaging; DTI, diffusion tensor imaging; DT, diffusion tensor; ICA, independent component analysis; ROIs, regions of interest; SC, structural connectivity; FC, functional connectivity; awFd, anatomically weighted functional dissimilarity; awFC, anatomically weighted functional connectivity; set of AFNI commands (3dMatch, 3dROIMaker, 3dNetCorr, 3dDWItoDT, 3dDWUncert, and 3dTrackID).

Figure 1 depicts how the *FATCAT-awFC* pipeline begins with the FATCAT and later transitions into the awFC method. From the FATCAT, functional connectivity (from the functional data) and “tract count” (from the structural data) are output. Subsequently, these two metrics are used as inputs for the awFC method.

Once “*tract count”* is output from the FATCAT approach (Taylor and Saad, 2013), a number of additional steps were performed using the awFC method to calculate *an improved* structural connectivity measure. These steps include: calculating the probabilities of structural connectivity, performing a Poisson-regression (to adjust for distance bias), computing and incorporating indirect (second-order) structural connectivity between ROI pairs (Bowman et al., 2012). Structural connectivity probabilities were estimated by calculating the 90th percentile of voxel-level counts connecting two ROIs, divided by the total streamlines leaving the ROI (Bowman et al., 2012). Next, the structural connectivity distance-bias was adjusted by fitting a zero-inflated Poisson regression model (Bowman et al., 2012). The Poisson regression was applied using: *log*(μ(*S*_*ij*_|*g*_*ij*_) = α_0_+α_1_*g*_*ij*)_, where *g*_*ij*_is the distance between each region pair, *S*_*ij*_is the unbiased number of tracts (Bowman et al., 2012). All possible second-order (indirect) connections were calculated using the equation: π_*ij*_ = max[π_*ij*_, max_*m*_(π_*im*_π_*mj*_)], where π is the probabilities of structural connectivity, i is the starting ROI, j is target ROI, and m is the third connection (Bowman et al., 2012). The greater connectivity value (between the direct and indirect connectivity) was taken to be the pathway between the connected ROIs (Bowman et al., 2012). Once all steps are performed, a structural connectivity metric is produced.

Functional connectivity was generated from the *FATCAT* pipeline and SC was produced from the *awFC method*. To combine FC and SC into a single metric, the dissimilarity metrics were first calculated (Bowman et al., 2012). FC and SC measure distinct aspects of brain connectivity, where FC measures the temporal correlation (using Pearson correlation), and SC measures the tract count between brain regions. Therefore, to generate a modality-independent comparison between structural and functional connectivity, the dissimilarity metric is used (Kriegeskorte et al., 2008). The structural dissimilarity (1 minus structural connectivity) and functional dissimilarity (1 minus functional connectivity) are multiplied to obtain the anatomically weighted functional dissimilarity. For ease of interpretation, the dissimilarity metric (a combined structural-functional measure) is transformed back to a correlation metric. This metric is known as the anatomically weighted functional connectivity, which is obtained by applying the equation: 1−|*awFd*| (Bowman et al., 2012).

### 3.6. Wilcoxon test and multiple comparisons adjustment

A Wilcoxon test was applied to determine whether there were any significant FC, SC, and awFC differences between children of mothers who experienced perinatal adversity compared to healthy controls. The R function, *“wilcox.test(),”* was employed to assess pair-wise differences in FC, SC, and awFC between the adversity and control groups for each ROI-pair within each RSN. False Discovery Rate (FDR) was used to correct for multiple comparisons (Waite and Campbell, 2006) *via* the function *“p.adjust()”* from the stats package in R (R Core Team, 2018). The significance level was set to *p*_*adj*_ < 0.05. Significant awFC differences between the control and adversity groups were visualized using boxplots, with the *“ggplot()”* function from the ggplot2 package (v.2.2.1) in R software (v.4.0.2). Finally, the effect size was calculated using Cohen’s *d, “cohen.d”* function from the effsize package (Tocrchiano, 2017) in R (R Core Team, 2018). A Cohen’s d between 0.5 > *d* > 0.2 is considered small, 0.8 > *d* > 0.5 moderate and *d* ≥ 0.8 large.

## 4. Results

### 4.1. Significant ROI-ROI pairs

**FIGURE 2:**
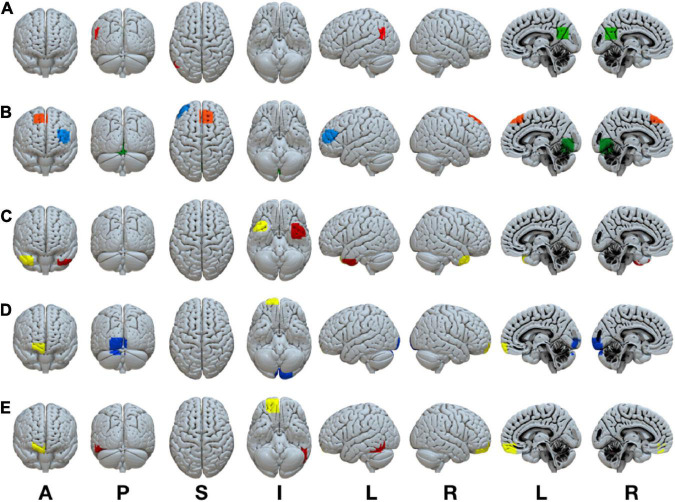
Statistically significant anatomically weighted functional connectivity group differences between brain regions are displayed for each network. Isolated brain regions were defined using the FATCAT command *3dROIMaker*. Each color represents a different ROI for each network **(A)** DMN, green ROI, posterior cingulate cortex, red ROI, left angular gyrus **(B)** FPN, blue ROI, left inferior frontal gyrus, orange ROI, right superior frontal gyrus, green ROI, lingual gyrus/cerebellum **(C)** LIM, yellow ROI, right superior temporal gyrus, red ROI, left superior temporal gyrus **(D)** VAN, yellow ROI, right anterior orbitofrontal gyrus, blue ROI, left lingual gyrus/cuneus **(E)** DAN, yellow ROI, right posterior orbitofrontal gyrus, red ROI, left inferior temporal gyrus. ROI, region of interest; DMN, default mode network; FPN, frontoparietal network; VAN, ventral attention network; DAN, dorsal attention network. Anatomical positions, A, anterior view; P, posterior view; S, superior view; I, inferior view; L, left view’ R, right view.

A complete listing of the ROIs in each RSN, their associated anatomical location, volume, and MNI coordinates is reported in Table 1. A visual representation of the ROIs that revealed significant connectivity differences between groups are shown in Figure 2.

**TABLE 1:** Complete listing of ROIs used in the study.

ROI no.	Anatomical names	MNI coordinates x, y, z			Volume (# of voxels)
**Default mode network**					
1	Medial frontal gyrus (MFG)	2	62	8	7
2	Posterior cingulate cortex (PCC)	−2	−62	24	5
3	Right angular gyrus (R-AG)	46	−58	28	6
4	Left angular gyrus (L-AG)	−46	−58	28	5
**Frontoparietal network**					
5	Left inferior frontal gyrus (L-IFG)	−38	38	12	12
6	Lingual gyrus/cerebellum (LG/CER)	−6	−74	−12	12
7	Right Superior frontal gyrus (R-SFG)	10	38	56	10
**Limbic network**					
8	Right posterior orbitofrontal gyrus (R-pOFG)	18	38	−24	12
9	Left posterior orbitofrontal gyrus (L-pOFG)	−42	30	−20	12
10	Right superior temporal gyrus (R-STG)	46	14	−44	11
11	Left superior temporal gyrus (L-STG)	−42	2	−52	12
12	Dorsolateral prefrontal cortex (DLPFC)	−2	46	32	12
**Ventral attention network**					
13	Left lingual gyrus/Cuneus–Lateral occipital cortex (L-LG/CU)	−14	−98	−16	20
14	Right orbitofrontal gyrus (R-aOFG)	14	70	−16	5
15	Right cerebellum (R-CER)	10	−66	44	20
**Dorsal attention network**					
16	Right anterior orbitofrontal gyrus (R-aOFG)	14	58	−20	9
17	Right posterior orbitofrontal gyrus (R-pOFG)	22	30	−16	14
18	Right middle frontal gyrus (R-MFG)	38	42	−16	14
19	L-Inferior temporal gyrus (L-ITG)	−50	−54	−16	14

Peak MNI coordinates and cluster sizes are given for these nineteen ROIs belonging to five resting-state networks: default mode, frontoparietal, limbic, ventral attention, and dorsal attention. ROIs were defined using FATCATs *3dROIMaker* command. ROI, region of interest; RSN, resting state network.

### 4.2. ROI-ROI awFC group differences

**FIGURE 3:**
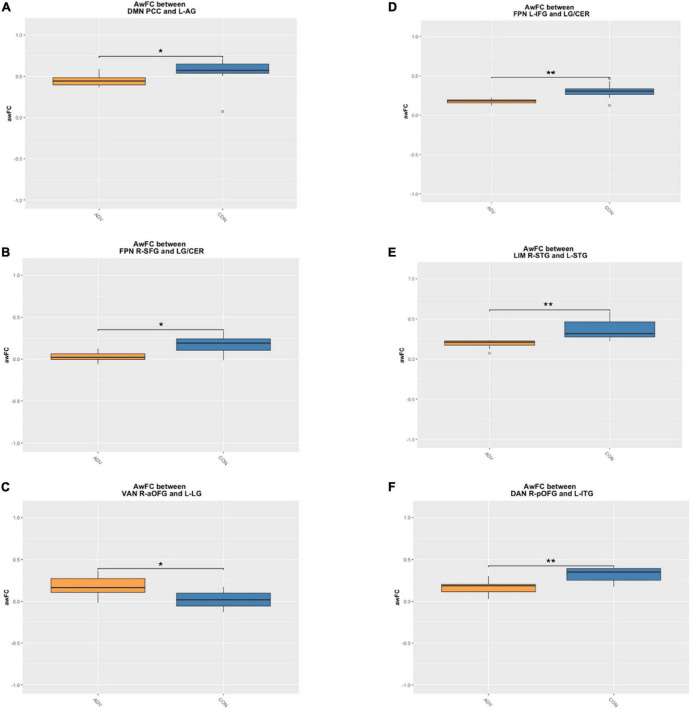
Boxplots demonstrate the anatomically weighted functional connectivity strength in children of mothers with a high adversity score (orange boxes) compared to children of mothers with a lower adversity score (blue boxes). Significant differences between HA and HC are shown within the **(A)** DMN between the PCC and L-AG **(B)** FPN between the L-IFG and the LG/CER **(C)** FPN between the R-SFG and the LG/CER **(D)** LIM between the R-STG and the L-STG **(E)** VAN between the R-aOFG and L-LG **(F)** DAN between the R-pOFG and L-ITG. adversity, children exposed to maternal adversity, control, healthy control group, The asterisks indicate a statistically significant difference in the ROI awFC between groups at *p < 0.05 and **p < 0.01. DMN, default mode network; FPN, frontoparietal network; LIM, limbic network; VAN, ventral attention network; DAN, dorsal attention network; L, left; R, right; IFG, inferior frontal gyrus; LG/CER, lingual gyrus/cerebellum; SFG, superior frontal gyrus; STG, superior temporal gyrus; aOFG, anterior orbitofrontal gyrus; LG, lingual gyrus; pOFG, posterior orbitofrontal gyrus; ITG, inferior temporal gyrus.

Children of mothers who experienced prenatal and/or postnatal adversity showed lower awFC in a number of ROI-pairs compared to healthy controls (Table 2). Specifically, in comparison to healthy controls, the adversity group exhibited lower awFC in (i) the DMN—between the PCC and the left angular gyrus (*p* = 0.0274), (ii) the FPN—between the left inferior frontal gyrus to the lingual gyrus/cerebellum (*p* = 0.0053), and between the right superior frontal gyrus and the lingual gyrus/cerebellum (*p* = 0.0274), (iii) the LIM—between the right superior temporal gyrus and the left superior temporal gyrus (*p* = 0.00149), and (iv) the DAN—between the right posterior orbitofrontal gyrus and the left inferior temporal gyrus (*p* = 0.032). Conversely, children exposed to prenatal and/or postnatal maternal adversity showed greater awFC in the VAN—between the right anterior orbitofrontal gyrus and the left lingual gyrus/cuneus (*p* = 0.00551). Group comparisons are shown in the boxplots in Figure 3. The multiple tests performed in this study were corrected for false discovery rate (FDR) to account for the potential inflation of Type I error due to multiple comparisons. FDR correction is a statistical method that adjusts the *p*-values based on the number of tests performed and the proportion of truly null hypotheses. This helps to control the rate of false positive findings and provides a more stringent criterion for declaring statistical significance. The results were considered statistically significant if the FDR-corrected *p*-value was less than 0.05.

**TABLE 2:** Group differences in connectivity from structural, functional, and anatomically weighted functional connectivity.

Start ROI	End ROI	SC *p*-value (FDR corrected)	FC *p*-value (FDR corrected)	awFC *p*-value (FDR corrected)	Cohen’s D
**Default mode network**					
PCC	L-AG	0.815	0.0274*	0.0274*	0.626 (medium)
**Frontoparietal network**					
L-IFG	LG/CER	**0.041**	0.006*	0.0053*	1.66 (large)
R-SFG	LG/CER	0.664	0.0274*	0.0274*	1.36 (large)
**Limbic network**					
R-STG	L-STG	0.095	0.002**	0.00149**	1.75 (large)
**Ventral attention network**					
R-aOFG	L-LG/CU	0.198	0.032*	0.032*	−1.27 (large)
**Dorsal attention network**					
R-pOFG	L-ITG	0.06	0.00551*	0.00551*	1.79 (large)

A Wilcoxon test was performed to reveal significant brain connectivity differences between children exposed to high maternal adversity (HA) and Healthy Control children. Significant awFC differences between children of mothers with high adversity compared to children of mothers with low adversity scores are shown, along with their corresponding structural and functional connectivity group differences. This table displays significance of group differences in connectivity values [bold identifies significant (*p* < 0.05)] of structural connectivity, functional connectivity, and awFC for each ROI-ROI pair. *Survives FDR (*q* < 0.05), **Survives FDR (*q* < 0.01). awFC, anatomically weighted functional connectivity; ROI, region of interest; SC, structural connectivity; FC, functional connectivity; PCC, posterior cingulate cortex; L-AG, left angular gyrus; L-IFG, left inferior frontal gyrus; LG/CER, lingual gyrus/cerebellum; R-SFG, right superior frontal gyrus; R-STG, right superior temporal gyrus; L-STG, left superior temporal gyrus; R-aOFG, right anterior orbitofrontal gyrus; L-LG/CU, left lingual gyrus/cuneus; R-pOFG, right posterior orbitofrontal gyrus; L-ITG, left inferior temporal gyrus.

### 4.3. ROI-ROI FC and SC group differences

In comparison to healthy controls, children exposed to perinatal maternal adversity exhibited FC differences in: (i) the DMN—between the PCC and the left angular gyrus (FC *p* = 0.0274, SC *p* = 0.815), (ii) the FPN—between the left inferior frontal gyrus to the lingual gyrus/cerebellum (FC *p* = 0.006, SC *p* = 0.041), and between the right superior frontal gyrus and the lingual gyrus/cerebellum (FC *p* = 0.0274, SC *p* = 0.664), (iii) the LIM—between the right superior temporal gyrus and the left superior temporal gyrus (FC *p* = 0.002, SC *p* = 0.095), (iv) the VAN—between the right anterior orbitofrontal gyrus to the left lingual gyrus/cuneus (FC *p* = 0.032, SC *p* = 0.198), and (v) the DAN—between the right posterior orbitofrontal gyrus and the left inferior temporal gyrus (FC *p* = 0.00551, SC *p* = 0.06). See Table 2 for a summary of the results.

Group differences in SC were observed in the FPN–between the left inferior frontal gyrus and the lingual gyrus/cerebellum. However, this difference did not survive the correction for multiple comparisons. The multiple comparisons made in the study were corrected using FDR correction to control for false positive findings and identify statistical significance with a stringent criterion of FDR-corrected *p*-value less than 0.05.

## 5. Discussion

Across biological scales (i.e., cellular formation, synaptic formation/pruning, myelination, macroscale network connectivity), there is a developmental progression from primary unimodal somatosensory/motor and visual regions to transmodal association cortices which support the emergence of complex cognition (Rakic et al., 1994; Cao et al., 2017; Casey et al., 2019; Paquola et al., 2019; Dong et al., 2022). Programmed neurodevelopmental events such as neurogenesis, myelination, and pruning drive the maturation of functional networks and brain structure (Grayson and Fair, 2017). Importantly, it is recognized that structural and functional development interact throughout development (Damoiseaux and Greicius, 2009; Suárez et al., 2020). Accordingly, in the present research, we identified differences in resting state networks, factoring in metrics of both FC and SC acquired from rs-fMRI and DTI imaging data. We hypothesized that the awFC method would provide a more sensitive approach to study network development in comparison to investigating SC and FC alone. The awFC method was used to explore differences in DMN, LIM, VAN, DAN, and FPN connectivity between children exposed to perinatal maternal adversity and a group of healthy control children. Our results suggest that in comparison to independent SC and FC analyses, the combined awFC method was more sensitive to differences in network connectivity within the LIM and FPN. We also found that children exposed to perinatal maternal adversity exhibited greater connectivity within the VAN in comparison to healthy controls.

### 5.1. Comparing awFC with SC and FC across regions of the FPN and LIM that distinguish between adversity and healthy control groups

The value of exploring differences in network connectivity using a fused structural-functional approach is reflected in our findings relating to the LIM and FPN. While analyses of group differences in SC and FC metrics separately identified network differences distinguishing the adversity from the control group, the connectivity differences were of greater significance when groups were contrasted using awFC data. These findings suggest that it may be important to consider the influence of SC when studying resting-state functional connectivity in developmental populations.

Using the awFC approach, we found that in comparison to healthy controls, children whose mothers experienced perinatal maternal adversity showed lower awFC in the FPN between the inferior frontal gyrus and cerebellum as well as the SFG and lingual gyrus. Our findings complement those from the maternal deprivation literature which consistently report that poor maternal mental health impacts the structural development of their offspring, especially in frontoparietal regions. For instance, postnatal depressive symptoms are negatively correlated with SFG thickness in children (Lebel et al., 2016). Similarly, fetal exposure to increasing levels of prenatal maternal stress is associated with reductions in SFG and lingual gyrus thickness (Davis et al., 2020). It has been reported that brain regions which show cortical thinning also exhibit weakened connectivity (Bullmore, 2019). Furthermore, research exploring the influence of SES on brain structure has revealed reduced cortical maturation (i.e., gray and white matter volume, integrity of WM tracts) in children exposed to low maternal SES compared to higher-SES children (Olson et al., 2021). These differences are hypothesized to result from changes in neuronal morphology, dendritic arborization, or stunted/suppressed synaptogenesis (Olson et al., 2021). The FPN is thought to play a role in executive functioning and cognitive control over emotion. Therefore, atypical FPN maturation may play a role in the widespread socioemotional, behavioral, and cognitive deficits reported among adversity exposed children (Hackman and Farah, 2009; Madigan et al., 2018; Pan et al., 2018; McLaughlin et al., 2019a,b; Oh et al., 2020; Priel et al., 2020). Evidently, as the literature suggests, maternal adversity impacts the structural neurodevelopment of offspring. Our study emphasizes that when examining developmental differences associated with adversity exposure, it is important to consider how structure interacts with function, rather than studying only one modality in isolation. Specifically, we demonstrate that SC can impact on estimates of FC between regions of the FPN. Importantly, attention to the sensitivity of analytical techniques when studying outcomes of adversity exposure may yield important information that helps characterize altered maturational brain changes.

The adversity group also displayed lower awFC in the LIM between the left and right superior temporal gyrus in comparison to healthy controls. Typically, interhemispheric connectivity strengthens with age (Smyser et al., 2010; Muetzel et al., 2016). In fact, RSN connectivity between homotopic regions is considered a ubiquitous feature of brain architecture (Salvador et al., 2005; Hermesdorf et al., 2016). Our results may point toward a delayed pattern of maturation for children in the adversity group exhibited lower awFC than the healthy control children. However, interpretations of delayed maturation should be taken with caution as we did not explore longitudinal developmental trajectories in this study. Alterations in homotopic connectivity have been identified in various psychiatric conditions, and specifically in the superior temporal gyrus amongst individuals with depression (Guo et al., 2013; Hermesdorf et al., 2016). Alike other brain regions, the superior temporal gyrus is implicated in a multitude of processes including language development, emotional functioning, and social cognition. As such, atypical development involving this region (as seen when comparing adversity exposed children to healthy controls) may increase the risk for poor outcomes. For instance, Noble et al. (2012) report that children raised in low SES environments demonstrated relatively smaller volumes in the left superior temporal gyrus, compared to their higher SES peers. It is suggested that protracted pruning allows for a period of prolonged plasticity which may be beneficial in higher SES environments (Noble et al., 2012). Further, there is consistent evidence that socioeconomically deprived environments and the lack of access to consistent and responsive caregiving are associated with differences in the development of emotion processing and language-supporting brain regions (Tomalski and Johnson, 2010; Tottenham, 2015). Similarly, our awFC results identified atypical development in the LIM among children exposed to perinatal maternal adversity. Crucially, the independent SC analysis did not identify significant differences in LIM connectivity between the adversity and control groups. However, by integrating SC in estimates of FC, the awFC method was able to capture important developmental changes related to interhemispheric connectivity in the LIM with a greater degree of sensitivity than FC alone.

Collectively, we report that when examining the FPN and LIM the awFC method was more sensitive than separate FC and SC analyses in detecting connectivity differences. We suggest that it is promising to see that the awFC method identified differences in higher-order cognitive and emotion networks as they are commonly reported neural targets of adversity exposure (Tomalski and Johnson, 2010; Tottenham, 2015; Chen and Baram, 2016) identified in existing unimodal imaging research.

### 5.2. Contrasting awFC between adversity and healthy control groups in the VAN

Greater FC in the VAN was identified for the adversity group compared to controls. This pattern was opposite to that observed in the other RSNs examined, where lower connectivity distinguished the adversity group from controls. Specifically, we found evidence of greater awFC within regions of the VAN (R aOFG and L LG/CU) among children whose mothers experienced perinatal adversity. Attention processing is associated with the engagement of two segregated functional networks—the DAN and the VAN—each subserving a specialized processing function (Corbetta and Shulman, 2002). The DAN is involved in goal-directed, top-down processing that maintains attention in the face of distracting stimuli (Corbetta et al., 2008). Conversely, the VAN is characterized as a stimulus-driven, bottom-up system that orients attention to stimuli outside of the current focus (Corbetta et al., 2008). Both networks interact to control which information is perceived and attended to Farrant and Uddin (2015). For instance, a granger causality analysis of task-based fMRI revealed that signals from the DAN to the VAN work to filter out unimportant distractor stimuli while signals from the VAN to the DAN interrupts attention maintained by the DAN in order to facilitate the reorientation of attention to newly salient stimuli (Wen et al., 2012). It has been observed that disruptions in the coupling of these networks results in attention deficits which underlie multiple psychopathologies (Suo et al., 2021). While these networks are well-characterized in adult neuroimaging studies, there is minimal research focusing on their developmental trajectory, with less exploration into changes in within-network connectivity as compared to between-network investigations (Farrant and Uddin, 2015). One study by Dong et al. (2022) found that children exhibiting low VAN functional connectivity displayed an accelerated profile of cortical maturation which resembles adolescence and adulthood organization. Dong et al. (2022) suggested that a decrease in VAN connectivity could signal the pruning of excessive/redundant connections to allow for more efficient information processing. However, both premature and delayed pruning may be consequential for future development. Furthermore, due to hierarchical nature of brain development, earlier maturing brain networks could shape the development of later developing cortical networks (Zeanah et al., 2011; Menon, 2013; Dong et al., 2022). The VAN is an intermediary network that coordinates signals between primary unimodal and the association cortices (Corbetta and Shulman, 2002; Konrad et al., 2005; Dong et al., 2022). As such, it is situated in a pivotal position to affect the development of multimodal association cortices which underlie higher-order cognitive functioning. Here, we observed greater connectivity in the VAN among the adversity group. While this finding will need to be replicated in a larger sample and explored across developmental timepoints, it suggests that altered VAN connectivity observed in association with maternal adversity may have knock-on effects that impact upon the consolidation of an adult-like functional cortical organization.

### 5.3. Structural connectivity and FC decoupling

In this study, we did not detect any differences in SC in the DMN, LIM, VAN, and DAN between healthy controls and children whose mothers experienced perinatal maternal adversity. Over development, synaptogenesis functions to increase the number of WM tracts while pruning decreases the number of these connections. It has been suggested that from age 2–7 years, the activity of these processes counterbalance one another resulting in a “plateau phase” (Levitt, 2003). Since the mean age of our sample was 7.63 years (*SD* = 0.66), it is possible that this feature might explain the lack of observable differences. Furthermore, WM maturation is driven by multiple processes. In this study, tract density was used as the metric for quantifying SC and therefore changes in myelination would not be detected by this approach. Accordingly, alterations SC may not be as easily identifiable during middle childhood in comparison to changes in FC. Developmental events such as pruning and myelination are also dependent on the network in question and vary in their timing and rate of change (Marsh et al., 2008; Walhovd et al., 2014). Inter-subject variability in maturational trajectories may also contribute to the incongruent SC and FC findings.

## 6. Limitations and future directions

Our research does have some important limitations which must be considered. First, owing to the loss of subjects due to movement during the scan, our sample size was small (17 participants). As such it will be important to replicate these findings in a larger sample. In addition, our study conceptualized maternal adversity as low SES and/or experiencing poor prenatal and/or postnatal maternal health (i.e., anxiety and/or depression). The field of adversity research continues to find optimal methods of measuring adversity exposure. The challenge may stem from the fact that adverse events co-occur, but each event can contribute to distinct neural outcomes (Sheridan and McLaughlin, 2014; McLaughlin and Sheridan, 2016). For example, living in low socioeconomic conditions is linked to an increased risk for abuse, lack of access to adequate healthcare, poor maternal mental health, etc. In a composite score, the specific effect of individual adverse experiences and their degree of severity would not be adequately represented in developmental outcomes. Furthermore, since our score measures maternal adversity across the perinatal period, we were unable to separate the independent impact of prenatal and postnatal exposures. Research efforts have suggested that prenatal and postnatal experiences do affect development in diverging ways, in addition to accounting for shared variance (O’Connor et al., 2002; Monk et al., 2012). Future investigations should continue to explore these relationships with consideration to different forms and severity of adversity exposure.

As this is the first study which has combined FC and SC using data fusion techniques to study developmental changes in middle childhood following perinatal maternal adversity exposures, comparing our results to the broader literature must be done with caution. Additional research using multimodal imaging would be beneficial for identifying whether accelerated vs. delayed maturation is dependent on imaging modality and level of analysis (e.g., ROI vs. network).

We used DTI-based tractography to identify white matter tracts. This method is highly prone to motion artifacts, inaccuracies and is unable to distinguish between the crossing, convergence, and divergence of fibers (Jbabdi and Johansen-Berg, 2011). We collected 3 DTI scans in each participant, permitting us to drop whole scans and retain some data, if movement was an issue. In addition, the number of vectors sampled was different in each of these scans (19, 20, and 21) giving us a total of 60 directions and improved capacity to resolve crossing fibers. Of note, it has been observed that diffusion spectrum imaging is better able to detect fibers and fiber crossings (Damoiseaux and Greicius, 2009) and therefore may provide a better suited technique for future studies.

## 7. Conclusion

To our knowledge, this is the first neuroimaging study which uses a combined data fusion structural-functional connectivity approach to explore the impact of perinatal maternal adversity on RSN development in middle childhood. We combine SC information from DTI with FC information from rs-fMRI to identify potential alterations in network connectivity within the VAN, DAN, DMN, FPN, and LIM upon perinatal adversity exposure. Our results indicated that there is a benefit of using an awFC approach as it is more sensitive in highlighting differences in connectivity in networks associated with higher-order cognitive and emotional processing (FPN and LIM) in comparison to independent FC and SC analyses. Furthermore, our awFC analysis suggests that poor maternal mental health and/or low SES during the perinatal period may be associated with greater awFC within the VAN. These results may suggest that it is possible for environmental perturbation in early life to recalibrate brain development in a way that facilitates survival within contexts of uncertainty and inconsistent caregiving.

## Data availability statement

The original contributions presented in this study are included in the article/supplementary material, further inquiries can be directed to the corresponding author.

## Ethics statement

The studies involving human participants were reviewed and approved by the ethics approval was obtained from Hamilton Integrated Research Ethics Board and the Douglas Mental Health University Institute. Written informed consent to participate in this study was provided by the participants’ legal guardian/next of kin.

## Author contributions

SA: methodology, analysis, and writing—draft. AS: writing—draft. HG: investigation and preliminary analysis. AH: investigation. AD: methodology, analysis, and writing review and editing. IP: investigation, and writing review and editing. MM and PS: conceptualization, and review and editing. RS: conceptualization review and editing. GH: conceptualization, funding, supervision, and writing review and editing. All authors contributed to the article and approved the submitted version.
